# Efficacy of an innovative PT program to enhance mobility in older Veterans with slow walking speed

**DOI:** 10.1093/geroni/igaf122.4344

**Published:** 2025-12-31

**Authors:** Jonathan Bean, Rebekah Harris, Elisa Ogawa, Elizabeth Rathje, Jennifer Brach, Paige Burns, Addie Middleton, Thomas Travison

**Affiliations:** Harvard Medical School, Boston, Massachusetts, United States; VA Boston Healthcare System, Boston, Massachusetts, United States; VA Boston Healthcare System, Boston, Massachusetts, United States; VA Boston Healthcare System, VA Boston Healthcare System, Massachusetts, United States; University of Pittsburgh, Pittsburgh, Pennsylvania, United States; VA Boston Healthcare System, VA Boston Healthcare System, Massachusetts, United States; Univ of Colorado, Anschutz Medical Campus, University of Colorado School of Medicine, Colorado, United States; Hebrew SeniorLife & Harvard Medical School, Hebrew SeniorLife, Massachusetts, United States

## Abstract

We investigated the efficacy of an innovative physical therapy program targeting mobility known as Live Long Walk Strong (LLWS). This was a single-blind randomized controlled trial conducted at an outpatient VA tertiary care center among community-dwelling middle-aged and older Veterans with slow walking speed. LLWS is an 8-week 10 (1-hour/session) session outpatient physical therapy program. It was designed to enhance mobility by targeting impairments linked to mobility decline and fostering long-term engagement in physical activity through coaching and behavior change strategies. LLWS treatment was compared to participants within a waitlist control group. Walking speed was the primary outcome and secondary outcomes included measures of mobility. We randomized 150 participants and experienced a 25% dropout rate, which was in part due to the COVID-19 pandemic shutdowns of human research (LLWS n = 56, control n = 58). We observed significantly faster (p<.05) walking speed (mean change 0.08±0.02 m/s) after the 8-week program, as well as significantly greater improvements in the Short Physical Performance Battery (mean change 0.95±0.32) and the Activity Measure for Post Acute Care (mean change 2.1±0.8) withing the LLWS group (all p < 0.05). These observed increases were consistent with large clinically meaningful differences. Our study demonstrates that LLWS enabled statistically significant and clinically meaningful gains in mobility among middle aged and older adult Veterans with slow walking speed, justifying its efficacy as a treatment for mobility limitations. Separate analyses of this clinical trial will evaluate long term outcomes and be better powered to evaluate changes in the attributes LLWS targets.

